# Amblyopia, Omaurosis, and the Extraction of Cataract

**Published:** 1866-11

**Authors:** Laurence Turnbull

**Affiliations:** Philadelphia


### SELECTIONS.

[CONCLUDED FROM PAGE 372.]

*Amblyopia, Omaurosis, and the Extraction of Cataract.* By LAURENCE TURNBULL, M. D., Philadelphia.

The "Clinical Remarks on a Case of Extraction of Cataract" occupy twenty-four pages. A few practical points we here marked, and first, as to simple cataract, if there is such a thing, "are those cases where we are unable to discover any affection of the deeper structures of the eye, and no break exists in the nervous apparatus. Only one eye should be operated upon at a time." The translator corroborates this from Wecker, who states that the elementary principles of prudence indicate sufficiently the impropriety of performing the operation of cataract on more than one eye at a time.

The opinion of some of Von Graefe's colleagues, that extreme age does not exert a particularly unfavorable influence on extraction, is most emphatically contradicted by his tables, which show, after the 65th, and particularly after the 70th year, a considerable falling off in the percentage of recoveries. A sunken position of the balls has been particularly dwelt upon as an unfavorable circumstance, in so far as it interferes with the mechanical execution of the operation. This is only so when it depends on general marasmus, and coupled with senile diminution in the diameter of the cornea. The tendency of iritis is decidedly greater in marasmic eyes, (increasing, as it does, in proportion to the age).

Taking all the circumstances into account, the general prognosis of flap extraction must be here essentially modified. According to his reckoning of 100 cases of flap extraction, 65 result favorably, by which he means the gaining an acuteness of vision of at least $\frac{1}{4}$; if more than 75 years of age, of at least 1-6. In 15 of the remaining 35, a favorable result is attained by a subsequent operation, consisting either in an operation for secondary cataract, or in an iridectomy with an operation for secondary cataract; of the 20 that now remain, about a third get at least vision enough to go about alone, (acuteness of vision 1-50 to 1-30,) a second third gain still less, and from 6 to 8 per cent of all eyes operated on remain or become entirely blind.

What advantage is to be gained by combining an iridectomy with flap-extraction? Does it ward off the danger of diffuse suppuration of the cornea? Not in the least. While it appears that iridectomy offers no protection against the occurrence of diffuse and partial suppuration, on the other hand, it does insure a more favorable course of things, and, to a certain extent, prevents the occurrence of iritis and prolapse of the iris. All so-called preparatory treatment is not only superfluous, but mischievous, unless indeed, there are special circumstances in the individual case requiring attention. It is sufficient to induce a gentle evacuation, by the use of castor oil or some other mild laxative, the day before the operation.

If we have a chance of previously watching the patient, it would be well to test the effect of a dose of morphine at least two days before the operation, in order to ascertain how the individual is affected by a drug we are so likely to subsequently employ, and which acts so differently in different cases. Use the upper section if we adopt the compressive bandage. In accordance with our usual practice, operate on the patient in bed. Special stress is laid on the proper management of the bandage after the operation. "The orbital hollow is first evenly packed with charpie, which has been picked over and put together in the form of small tufts, the whole being secured by a single turn of a snug-fitting flannel bandage passing over one eye. This is held in place by another single turn around the forehead, the first half of which comes before, the other half after the turn, passing over the eye. The middle portion of the bandage passing over the eye is knit of cotton, and not of flannel. Tolerably firm pressure must be made during the first few hours, and then gradually relaxed, in order not to hinder the escape of liquid secretions."

The author gives the details of a case of a female, aged 64, which we cannot follow, but sufficient for us to give the result after the operation. The entire corneal wound becomes infiltrated with opaque yellow matter, to the extent of nearly 1''', and evidently throughout its entire thickness. The whole corneal flap has a yellowish, sodden appearance. Through its upper third alone is the iris visible, and the reestablishment of the anterior chamber evident. The cut edges are not in contact. The suppuration is not from the iris, but from the cornea. What can we do that will tend to limit the suppuration? Shall ice-cold applications be made? No. Application of leeches? No. Shall venesection be performed? No. What remains to be done? The bandage changed every three hours, subsequently at longer intervals, in case the suppuration shows the desired diminution. Between the applications of the bandage, camomile fomentations at a temperature of 95 degrees, are to be used on the lids. The diet to be bouillon, and to drink milk. The operation was performed, we should judge, on the 4th of January. On the 9th, the warm fomentations were omitted, and atropine to be instilled.

January 20. The progress of the case has been as favorable as could possibly have been anticipated. The purulent infiltration in the vicinity of the wound has become more consolidated, and will leave a cicatrix about ¾''' in breadth. A tissue in process of organization may be seen to extend from the wound to the pupil, indicating the course of the previous suppuration. The effect of the atropine may be seen in an enlargement of the pupil upward—artificial pupil entirely filled and contracted for a subsequent iridectomy; and this he considers due to the constrictive bandage kept up for five days after the date of the last record. The results would have been more favorable if the bandage had been used earlier.

In concluding our notice of Dr. Derby's translation, we most heartily thank him for his labor of love for our benefit, for it is a most valuable addition to our knowledge of the subjects treated of, more especially that part of it on the difficulties and dangers of all the forms of extraction of cataract. The customary mode is to report only successful cases, and so to give the impression that in the great majority of instances it is a successful operation. But our experience of the last twenty years has demonstrated the falsity of many of the reports in regard to this operation, and indeed of all operations for the removal of cataract. We find that, since 1863, Graefe has modified his operation, and his new method of extraction he calls "the modified linear extraction." According to Dr. A. D. Williams, "since June, 1865, he has practised this new operation exclusively in all cases of adults where the cataract is hard." A full translation of this method, by Dr. Samelson, will be found in course of publication, with illustrations, in the *London Ophthalmic Review*, which we before referred to; but we, in our short notice, prefer the letter of an American physician, who gives a more concise description of the *modus operandi* and results. The patient lies on a sofa or couch, as usual.

Chloroform may be administered or not, as may be thought best by the operator, or desired by the patient.

"A strong wire speculum is introduced, which holds the lids wide apart. The eye is fixed with a forceps by fastening the conjunctiva near the lower margin of the cornea, and can be turned downward or in any desired direction. When thus depressed and held by the forceps, the eye is ready for the incision. The knife used is very small, varying from one to one and a quarter inches in length, and from one and a half to two lines in width in its widest part. The point is very fine and sharp, and passes almost without resistance through the cornea. The incision is made directly upward, the eye, of course being held in proper position. The sclerotica is punctured as far back as possible, so as still to enter the anterior chamber directly in front of the outer margin of the iris. The knife is now carried forward in the direction of the centre of the dilated pupil, until it passes beyond that centre, when it is turned upward close in front of the iris, on the opposite side, where the *counter*-puncture is effected through the sclerotica. It is now held close to the anterior surface of the iris, and by a gentle, sawing motion the flap is completed. When the section of the sclerotica is accomplished, the edge of the knife is turned directly forward, so as not to make too large a conjunctival flap. The length of the wound should embrace about one-third of the circumference of the eye, parallel with, but behind the margin of the cornea. The conjunctival flap is now moved out of the way by gently laying it back with a pair of forceps toward the cornea. The iris generally prolapses, and now lies perfectly naked in the wound. Simple iridectomy, in the usual, well known way, is next made, and must be large, extending from one end of the incision to the other. The large iridectomy facilitates very much the exit of the cataract, by making the opening through which it has to pass out freer. Now comes the incision of the anterior capsule, which is done in a peculiar way. For this purpose, a common hook, slightly bent so as to facilitate its introduction into the anterior chamber, is used. It is passed in front of the lens, till it comes nearly opposite the lower margin of the expanded pupil, when by rotation, the point is brought in contact with the capsule. Then by gently drawing it upward and inward, the capsule is divided entirely to the upper and inward margin of the lens, terminating at the edge of the artificial pupil. A second slit is now made, starting from the original point, and terminating at the upper and outer margin of the cataract and the corresponding edge of the new pupil. In this way the capsule is opened in the form of a V, with the base upward, so that every possible obstacle to the escape of the cataractous lens is removed. Now, how is the lens to be extracted? He makes use of a pretty large scoop, and bent at a more accurate angle than that of Daviel, for convenience of manipulation. The posteral lip of the scleral wound is now pressed gently downward and inward, so as to bring it rather beneath the upper edge of the lens, and thus allow the cataract to slip out. Should this not succeed, he makes a 'sliding manœuvre' from right to left, or *vice versa*, along the posterior lip from one end to the other, passing it inward at the same time, as much as is advisable. This simple movement frequently resulted in loosening the cataract, and causing it to escape. If this, in turn, does not succeed, he lays down the scoop, and takes up a small hook, made expressly for the purpose, and introduces it through the capsular opening at the upper margin, passes it carefully behind the lens but within the capsule, till its point reaches near the lower edge of the same. The hook, of course, introduced with the plane of the point parallel to the capsule, is now turned with the point forward, and pressed into the hard substance of the lens. By very *slight* traction the cataract is drawn out. It is to be supposed that one or the other of these three manœuvres will be successful. Up to the present time I have not witnessed a single operation when they all failed. From my observations the hook will have to be used in about half the cases. As in all other operations for extraction, these manipulations must be made with gentleness and caution, and never in a hurry, or with force. These delicate precautions are necessary to avoid the rupture of the hyaloid membrane and escape of vitreous humor. After the hard nucleus is removed, the remaining soft cortical substance is induced to escape by gently rubbing the lower lid over the cornea a few times, from below upward, as in ordinary flap extractions. The wound in the sclerotica is now cleared carefully of all particles of lens, or coagula of blood, so that the coaptation may be perfect. The flap of conjunctiva is now turned back into its natural position, and nicely adapted with the forceps, so as to cover the wound in the sclerotica. The eye is at last closed, the orbital cavity filled up with charpie, and over this the usual bandage, moderately tight, is applied. I should have mentioned before, that the speculum and fixation forceps are removed immediately after the exit of the nucleus, or bulb of the cataract. The patient is kept quietly in bed, and free from all excitement, and not allowed to talk or chew anything hard. The diet is restricted to rather a minimum quantity of fluid articles, and the patient is not allowed to rise up, unless it is absolutely necessary to answer the calls nature. Some six or seven hours after the operation the eye is opened, the charpie replaced by fresh, and the bandage is taken off twice, and atropia instilled into the eye, and this is repeated each day. By keeping the eye bound up several days, the possible springing of the wound is avoided, an accident by no means uncommon after flap extractions. Only one instance of this kind is reported, after the operation by Graefe's method. The operations are all made down stairs, so that the patient must be led up two or three flight of stairs to their rooms. On an average, patients are discharged, after the operation, in from ten to twelve days, and some even inside of a week. On the second day they are allowed to sit up if they wish. I see persons here who are operated on Friday, and on Monday are taken down to the lecture-room to be presented to the class, and again walk up the stairs to their rooms. Such a thing would hardly be allowed within ten days after an ordinary extraction. The wound in a sclerotica heals first by intention, and in a remarkably short time. The conjunctival flap unites very soon, by means of its sub-conjunctival tissue to the episclera, and thus completely closes the wound in the sclerotica. A *cystoid cycatrix*, which occurs so often after iridectomy in glaucomatous eyes, has not yet been observed after this modified form of linear extraction. By cystoid cycatrix is meant those instances where the cut in the sclerotica does not heal, but allows the aqueous humor to escape through it under the conjunctiva that has united over it—thus forming a bladder or little cyst—hence the name. This form of cicatrization after iridectomy for glaucoma, is not owing to tne fact the *incision is made in the sclerotica.* The cause is to be sought in the nature of the disease, especially the unnatural tension of glaucomatous eyes, which predisposes to healing by such a process.

"The reaction after Graefe's new method, is very inconsiderable indeed. The conjunctiva, it is true, reddens smartly from the incision and from the irritation produced by the fixation forceps, but the cornea and sclerotica only exceptionally, take on even a slight degree of inflammation. As a rule, little or no pain follows the operation, and no constitutional disturbance. Some stress has been laid on the question as to who shall hold the forceps during the different stages of the operation. This, I think, may be left to option and convenience of the operator. He may fix the eye himself, or entrust it to a safe and reliable assistant.

"In the brief account which I have given of the 'modified linear extraction'—a method that promises, sooner or later, to come into general use—I have confined myself to a statement of the different steps in the *normal* operation, and have avoided purely theoretical questions, Graefe, in the article above excited, gives the results of sixty-nine cases operated by his method. Of these, none were completely lost. There were sixty-two perfect, and seven imperfect results. Among the latter were six who could see comparatively well, and two, at the time of his writing, (Aug. 1865,) stood a fair chance to get better vision without a second operation. Four will have to be operated on for secondary cataracts, an occurrence not at all uncommon after all cataract operations. In the seventh case the sight was very imperfect, but there was a tolerable prospect of improvement by subsequent removal of the opaque capsule."

In regard to this modification of Von Graefe's, it must be tested by numerous operators before a true verdict can be given. Dr. Wecker speaks in his recent work in the most favorable manner of *his method*, which is the extraction of cataract, together with its capsule, without laceration, under the influence of ether, which anæsthetic he prefers to chloroform.—*Med. and Surg. Reporter.*

